# A metabolomics-based strategy for identification of gene targets for phenotype improvement and its application to 1-butanol tolerance in *Saccharomyces cerevisiae*

**DOI:** 10.1186/s13068-015-0330-z

**Published:** 2015-09-15

**Authors:** Shao Thing Teoh, Sastia Putri, Yukio Mukai, Takeshi Bamba, Eiichiro Fukusaki

**Affiliations:** Department of Biotechnology, Graduate School of Engineering, Osaka University, 2-1 Yamadaoka, Suita, Osaka, 565-0871 Japan; Department of Bioscience, Nagahama Institute of Bio-Science and Technology, 1266 Tamura, Nagahama, Shiga 526-0829 Japan

**Keywords:** Metabolomics, Semi-rational strain engineering, Phenotype improvement, 1-Butanol tolerance, *Saccharomyces cerevisiae*, Regression model, Orthogonal projections to latent structures

## Abstract

**Background:**

Traditional approaches to phenotype improvement include rational selection of genes for modification, and probability-driven processes such as laboratory evolution or random mutagenesis. A promising middle-ground approach is semi-rational engineering, where genetic modification targets are inferred from system-wide comparison of strains. Here, we have applied a metabolomics-based, semi-rational strategy of phenotype improvement to 1-butanol tolerance in *Saccharomyces cerevisiae*.

**Results:**

Nineteen yeast single-deletion mutant strains with varying growth rates under 1-butanol stress were subjected to non-targeted metabolome analysis by GC/MS, and a regression model was constructed using metabolite peak intensities as predictors and stress growth rates as the response. From this model, metabolites positively and negatively correlated with growth rate were identified including threonine and citric acid. Based on the assumption that these metabolites were linked to 1-butanol tolerance, new deletion strains accumulating higher threonine or lower citric acid were selected and subjected to tolerance measurement and metabolome analysis. The new strains exhibiting the predicted changes in metabolite levels also displayed significantly higher growth rate under stress over the control strain, thus validating the link between these metabolites and 1-butanol tolerance.

**Conclusions:**

A strategy for semi-rational phenotype improvement using metabolomics was proposed and applied to the 1-butanol tolerance of *S. cerevisiae*. Metabolites correlated with growth rate under 1-butanol stress were identified, and new mutant strains showing higher growth rate under stress could be selected based on these metabolites. The results demonstrate the potential of metabolomics in semi-rational strain engineering.

**Electronic supplementary material:**

The online version of this article (doi:10.1186/s13068-015-0330-z) contains supplementary material, which is available to authorized users.

## Background

Developments in recombinant DNA technology and bioprocess engineering have enabled microbial production of a wide variety of useful compounds including food products, pharmaceuticals and biofuels. However, the tailoring of host microorganisms for competitive industrial-scale production remains a challenging task. Growth rate, production rate, yield, side product formation and tolerance to various stresses encountered in a bioproduction process are some of the phenotypic traits that affect production and demand improvement. Traditionally, strain engineering has centered on rational approaches, whereby gene targets for disruption or overexpression are selected based on knowledge of genetic mechanisms and metabolic pathways that govern the phenotype [1, 2]. However, elucidation of these mechanisms involves complicated and time-consuming classical genetics studies, molecular biology methods and biochemical analyses, and there is a possibility that information from such prerequisite investigations may not be available for a less-studied phenotype. Due to this requirement for prior knowledge, rational engineering will always lag behind efforts to elucidate the underlying mechanisms. In addition, it has been suggested that improvement of complex phenotypes such as tolerance, which are governed by multiple, interconnected mechanisms, would be difficult due to the large number of gene target choices and the various possible interactions between them [3, 4].

At the opposite end of the scale, strategies such as laboratory evolution [5], random mutagenesis [6], transcription factor mutagenesis [3], or genome shuffling [7] rely on completely random processes to produce strains with improved phenotypes, which bypasses the need for any elucidation of the mechanisms involved [1]. However, these approaches offer no hints as to which genes are important; therefore, further improvement of the phenotype is difficult [1]. Another issue is that a screening step is often required to recover the improved strains, which may be tedious or impractical depending on the phenotype [4]; for example, to obtain improved ethanol-fermenting strains of *Kluyveromyces marxianus*, fermentation performances of 25,200 strains were individually tested following mutagenesis by UV irradiation [8].

With the advent of high-throughput, comprehensive ‘omics’ analytical technologies, selection of gene targets based on comparison of genomes or transcriptomes—termed here the ‘semi-rational’ approach—has become available [2, 9–11]. An advantage of the semi-rational strategy is that rather than being limited by lack of prior knowledge, the results actually contribute information on which genes are important to the phenotype, which facilitates subsequent rational strain modification to achieve further improvement [12]. For example, examination of the yeast transcriptomic response to ethanol stress [13] and phenotypic analysis of gene knockout libraries [14] have yielded genes important for ethanol tolerance. Strategies using laboratory evolution followed by genomic analysis and comparison have also yielded productive gene targets, e.g., for improving ethanol tolerance of yeast [12] or isobutanol tolerance of *Escherichia coli* [15]. The advantages and weaknesses of the three approaches—rational, semi-rational and random—are summarized in Table 1.

**Table 1:** Rational, semi-rational and random approaches in strain engineering

Rational	Semi-rational	Random
Gene modifications based on knowledge of genetic mechanisms and metabolic pathways governing the phenotype	Genetic modifications based on system-level comparison between strains, conditions, etc.	Genetic changes driven by random processes
Ex. Expression of heat-shock proteins or alcohol efflux pumps for heat or alcohol stress tolerance, respectively	Ex. Genome-wide or transcriptome-wide comparison	Ex. Laboratory evolution, induced transcription factor mutagenesis
Dependence on availability of prior knowledge	Not limited by lack of prior knowledge	Not limited by lack of prior knowledge
Results contribute additional information on relevant or important genes	Results contribute additional information on relevant or important genes	Results by themselves do not provide information on which genes are relevant/important
Elucidation of mechanisms is complicated and time-consuming	High-throughput, comprehensive ‘omics’ analytical technology is required	Screening step often required to recover the improved strains

The flow of information between gene and phenotype is subject to interventions such as post-transcriptional regulation or protein post-translational modifications. Because of this, differences at the gene or transcript levels as revealed by genomics or transcriptomics, respectively, may fail to be reflected in the final phenotype [16]. On the other hand, metabolomics—the technological field concerned with the comprehensive analysis of intracellular metabolites (the ‘metabolome’)—interrogates the cell at a level closer to the phenotype, and has shown utility in both basic and applied research [17, 18]. Unlike transcripts and proteins, which are considered media in the flow of genetic information, metabolites are the end products of cellular regulatory processes, and their levels are regarded as the ultimate response of biological systems to genetic or environmental changes [19, 20]. Thus changes in the metabolome are expected to show a high degree of correlation with the objective phenotype (i.e., tolerance). The link between metabolome and phenotype is direct and less subject to interventions, and comparative metabolomics may hence provide many relevant ‘hits’ complementary to those obtained by genomics or transcriptomics.

A unique feature of metabolomics data is that it is particularly suited for constructing quantitative models. Measurement of metabolites is highly reproducible and has good dynamic range, enabling metabolite levels to take the role of truly quantitative predictor variables instead of binary (‘on/off’) ones. The tight coupling with phenotype also means that when the phenotype is quantitative, so is the variance in the associated metabolite levels. While transcriptomics data is often simply used to compare or classify different classes of samples (e.g., ‘normal vs disease’, ‘control vs treated’, ‘sensitive vs tolerant’), metabolomics data has been used to model linear relationships between metabolite levels and quantitative phenotypes such as vertebrate developmental stage [21] and yeast replicative lifespan [22].

Further, detailed examination of such models can reveal the metabolites and associated metabolic pathways related to the phenotype, which may suggest gene targets for phenotype modification. One such example can be found in [22], where lifespan-related yeast mutants were analyzed by metabolomics, a regression model constructed from the metabolite profiles, and gene deletions were suggested based on important metabolites indicated by the model—a large percentage of which successfully modified replicative lifespan in the predicted manner, despite the genes’ actual mechanistic role in mediating lifespan being unknown. It was concluded that metabolomics can be a powerful tool to guide the semi-rational selection of gene targets for phenotype improvement, and we propose that this approach will also be effective in the improvement of phenotypes important in a microbial bioproduction process (growth rate, productivity, tolerance).

The objective of this study was to demonstrate the usefulness of metabolomics in semi-rational strain engineering, by identifying gene targets using only information inferred from metabolomics data. Here, we have used 1-butanol tolerance of *Saccharomyces cerevisiae* as the test phenotype. Medium-chain alcohols such as 1-butanol are more toxic than ethanol [1] and affect cells through disruption of the cell membrane, interruption of cellular processes such as energy generation and nutrient transport, protein denaturation, oxidative damage to DNA and lipids, and RNA unfolding and degradation [23]. The measurable effects of stress include reductions in growth rate, maximum cell density, substrate uptake rate, product formation rate, yield, etc., and tolerance broadly refers to the cells’ ability to resist these effects. Growth inhibition is suggested as one bottleneck in the microbial production of higher alcohols such as 1-butanol [24], and in this study, we focused on the maximal growth rate under stress as the measure of 1-butanol tolerance. *Saccharomyces cerevisiae* was selected as the microbial platform for this proof-of-concept as it is (1) a model organism for which a large resource of genetic and physiological information is available; (2) amenable to genetic modification using well-established techniques; and (3) a potential host organism for industrial biofuel production [25].

**Fig. 1:**
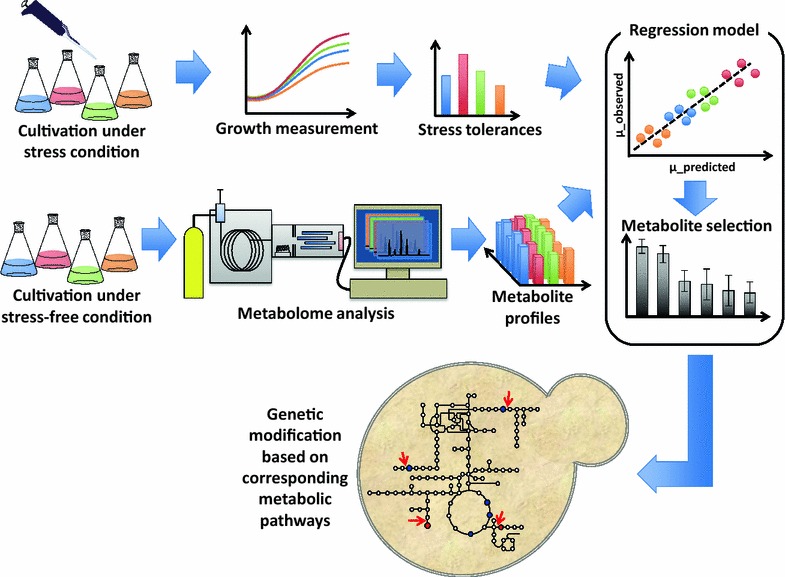
Metabolomics-based strategy of phenotype improvement. Various mutant strains of *S. cerevisiae* are cultivated in the presence of 1-butanol stress to measure their tolerances. These strains are also cultivated under the non-stress condition for metabolome analysis. The metabolomics and tolerance data are combined to construct a regression model, which provides information on which metabolites are strongly associated with tolerance. On the assumption that these metabolites have a causal relationship with tolerance, the corresponding metabolic pathways are examined, and gene deletion targets predicted to modify metabolite pools in the direction associated with increased tolerance are then identified. Finally, new deletion strains are obtained, and their tolerances and metabolite pools are measured to validate the model predictions

An overview of the strategy followed in this study is shown in Fig. 1. Firstly, various mutant strains of *S. cerevisiae* are cultivated in the presence of 1-butanol to measure their growth rates under stress. These strains are also cultivated under the non-stress condition for metabolome analysis. The metabolomics and growth rate data are combined to construct a regression model, which provides information on which metabolites are strongly associated with the growth rate under stress. On the assumption that these metabolites have a causal relationship with the stress growth rate, the corresponding metabolic pathways are examined, and gene deletion targets predicted to modify metabolite pools in the direction associated with higher growth rates under stress are then identified. Finally, new deletion strains are obtained, and their stress growth rates and metabolite pools are measured to validate the model predictions.

## Results and discussion

### Strains selection and 1-butanol tolerance measurement

We started with a set of yeast mutant strains based on the BY4742 genetic background, each a single disruption mutant of a gene annotated as ‘transcription factor’ in the *Saccharomyces* Genome Database [26]. Transcription factor mutants were chosen for this strategy because they are more likely to have differing fitness phenotypes under environmental perturbation [27], which would yield a range of different growth rates under 1-butanol stress. Also, the deletion of a single transcription factor may affect the expression of multiple genes, providing a global perturbation to the metabolite profile; a set of transcription factor mutants, therefore, is expected to contain a variety of different metabolite profiles. Both features were considered essential for obtaining a good regression of stress growth rate on metabolite data.

A preliminary round of 1-butanol tolerance measurements was carried out to select suitable strains for metabolome analysis. One hundred and seven (107) strains annotated with ‘transcription factor’ and one or more of the keywords {‘metabolism’, ‘synthesis’, ‘biosynthesis’, ‘catabolism’, ‘stress’, ‘response’, ‘tolerance’, ‘resistance’} (see Additional file 1: Table S1) were chosen and their specific growth rates with and without 1 % (v/v) 1-butanol stress were measured. From the preliminary measurements (Additional file 1: Table S2), strains were selected for further study based on the following criteria:

1. Deletion strains of metabolism-related transcription factors (according to gene description in Saccharomyces Genome Database, see annotation ‘II’ in Additional file 1: Table S1; 33 strains fulfilled this criteria).
2. Stress growth rates cover a range of different values from high to low.
3. Non-stress growth rates are similar to parental type BY4742 (growth rate value difference from BY4742 within one standard deviation of preliminary measurement values; 86 strains fulfilled this criteria); this is to exclude growth-rate related effects on the non-stress metabolomes to be analyzed.

**Fig. 2:**
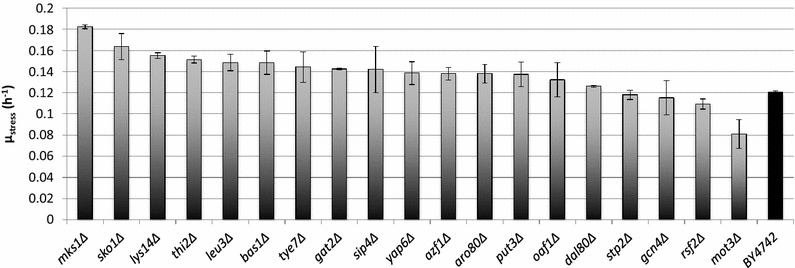
1-Butanol tolerance measurements for 19 selected strains. Specific growth rates for selected strains under 1.5 % (v/v) 1-butanol stress condition (*µ*_stress_). The BY4742 parental strain is also included for reference. *Columns* represent average values and *error bars* represent standard deviations from duplicate measurements. The measurement values are found in Additional file 1: Table S3

Using these criteria, 19 strains were selected (Table 2). A new round of stress growth rate measurement, with the aim of providing tolerance values for regression modeling, was then repeated for these strains. This time, 1.5 % (v/v) 1-butanol was used for the stress condition in order to emphasize differences between the strains, and the average of duplicate (*n* = 2) measurements was taken for each strain (Fig. 2). The measurement values ranged from 0.0810 h^−1^ (*mot3*∆) to 0.182 h^−1^ (*mks1*∆) with a median value of 0.139 h^−1^. Following the measurements, the maximum (mid-exponential) specific growth rate under stress, *µ*_stress_ was taken as the indicator of 1-butanol tolerance for each strain.

**Table 2:** Strains selected for metabolome analysis and regression modeling

Strain	Description in *Saccharomyces* genome database
*aro80∆*	Zinc-finger transcriptional activator of the Zn2Cys6 family; activates transcription of aromatic amino acid catabolic genes in the presence of aromatic amino acids
*azf1∆*	Zinc-finger transcription factor, involved in induction of CLN3 transcription in response to glucose; genetic and physical interactions indicate a possible role in mitochondrial transcription or genome maintenance
*bas1∆*	Myb-related transcription factor involved in regulating basal and induced expression of genes of the purine and histidine biosynthesis pathways; also involved in regulation of meiotic recombination at specific genes
*dal80∆*	Negative regulator of genes in multiple nitrogen degradation pathways; expression is regulated by nitrogen levels and by Gln3p; member of the GATA-binding family, forms homodimers and heterodimers with Deh1p
*gat2∆*	Protein containing GATA family zinc-finger motifs; similar to Gln3p and Dal80p; expression repressed by leucine
*gcn4∆*	Basic leucine zipper (bZIP) transcriptional activator of amino acid biosynthetic genes in response to amino acid starvation; expression is tightly regulated at both the transcriptional and translational levels
*leu3∆*	Zinc-knuckle transcription factor, repressor and activator; regulates genes involved in branched chain amino acid biosynthesis and ammonia assimilation; acts as a repressor in leucine-replete conditions and as an activator in the presence of alpha-isopropylmalate, an intermediate in leucine biosynthesis that accumulates during leucine starvation
*lys14∆*	Transcriptional activator involved in regulation of genes of the lysine biosynthesis pathway; requires 2-aminoadipate semialdehyde as co-inducer
*mks1∆*	Pleiotropic negative transcriptional regulator involved in Ras-CAMP and lysine biosynthetic pathways and nitrogen regulation; involved in retrograde (RTG) mitochondria-to-nucleus signaling
*mot3∆*	Transcriptional repressor and activator with two C2-H2 zinc fingers; involved in repression of a subset of hypoxic genes by Rox1p, repression of several DAN/TIR genes during aerobic growth, and repression of ergosterol biosynthetic genes in response to hyperosmotic stress; contributes to recruitment of the Tup1p-Cyc8p general repressor to promoters; involved in positive transcriptional regulation of CWP2 and other genes; can form the [MOT3+] prion
*oaf1∆*	Oleate-activated transcription factor, acts alone and as a heterodimer with Pip2p; activates genes involved in beta-oxidation of fatty acids and peroxisome organization and biogenesis
*put3∆*	Transcriptional activator of proline utilization genes, constitutively binds PUT1 and PUT2 promoter sequences as a dimer and undergoes a conformational change to form the active state; differentially phosphorylated in the presence of different nitrogen sources; has a Zn(2)-Cys(6) binuclear cluster domain
*rsf2∆*	Zinc-finger protein involved in transcriptional control of both nuclear and mitochondrial genes, many of which specify products required for glycerol-based growth, respiration, and other functions
*sip4∆*	C6 zinc cluster transcriptional activator that binds to the carbon source-responsive element (CSRE) of gluconeogenic genes; involved in the positive regulation of gluconeogenesis; regulated by Snf1p protein kinase; localized to the nucleus
sko1∆*	Basic leucine zipper transcription factor of the ATF/CREB family; forms a complex with Tup1p and Cyc8p to both activate and repress transcription; cytosolic and nuclear protein involved in osmotic and oxidative stress responses
*stp2∆*	Transcription factor, activated by proteolytic processing in response to signals from the SPS sensor system for external amino acids; activates transcription of amino acid permease genes
*thi2∆*	Transcriptional activator of thiamine biosynthetic genes; interacts with regulatory factor Thi3p to control expression of thiamine biosynthetic genes with respect to thiamine availability; acts together with Pdc2p to respond to thiaminediphosphate demand, possibly as related to carbon source availability; zinc-finger protein of the Zn(II)2Cys6 type
*tye7∆*	Serine-rich protein that contains a basic-helix-loop-helix (bHLH) DNA binding motif; binds E-boxes of glycolytic genes and contributes to their activation; may function as a transcriptional activator in Ty1-mediated gene expression
*yap6∆*	Basic leucine zipper (bZIP) transcription factor; physically interacts with the Tup1-Cyc8 complex and recruits Tup1p to its targets; overexpression increases sodium and lithium tolerance; computational analysis suggests a role in regulation of expression of genes involved in carbohydrate metabolism

### Metabolome analysis using gas chromatography coupled to mass spectrometry (GC/MS)

Metabolomics-based comparison of strains growing at different rates is complicated by the growth-dependency of the metabolome [28]. An alternative was to measure the non-stress metabolomes of the strains and use them for the modeling of growth rates (i.e., ‘tolerance’) under stress. It has been suggested that intracellular metabolite concentrations may change to compensate for the effects of genetic perturbation and maintain metabolic fluxes, e.g., growth rates [29]. We observe that while this homeostasis may be achieved under normal growth conditions, the perturbation may exceed the cells’ compensation ability under conditions of stress (e.g., 1-butanol) and hence the growth rate may change. This suggests that differences in metabolomes measured under normal, non-stress conditions may be used to predict growth rate phenotypes under stress conditions, and based on this we decided to sample the strains’ metabolomes under the non-stress condition.

**Fig. 3:**
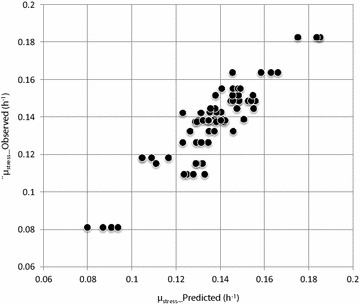
OPLS model observed vs predicted plot. Response variable (tolerance) values calculated from metabolite data of each sample according to the model (*µ*_stress__Predicted), plotted against the corresponding strain’s average measured tolerance (*µ*_stress__Observed). *N* = 4 samples were prepared for each strain in the metabolome analysis

Although it is estimated that there are only about 600 low molecular weight metabolites in *S. cerevisiae* compared to its 6000 or so genes [29], the chemical diversity of these metabolites makes comprehensive measurement with a single analytical platform currently impossible. Therefore, in this study the scope of analysis was limited to only hydrophilic primary metabolites, since there are established protocols for their extraction, derivatization and analysis [30] and information from these metabolites has been shown to be useful for the prediction of cellular physiological states and phenotypes (for example zebrafish developmental stage [21] and yeast replicative lifespan [22]). Gas chromatography coupled to mass spectrometry (GC/MS) was selected as our analytical platform as it allows reproducible measurement, acquisition of a large number of compound peaks from a biological sample, and straightforward peak identification using retention index (RI) and mass spectra by matching to an available reference library [31]. These advantages enabled us to employ untargeted analysis and retain all detectable, reliable peaks to use as predictor variables for regression modeling.

Following tolerance measurement, we subjected the 19 strains above to metabolome analysis. Since between-strain comparison was the goal of the analysis, absolute quantification was not necessary and hence we aimed to simply relative levels of metabolites for each strain. Four biological replicates (n = 4) were prepared for each strain, and samples were cultivated, prepared and analyzed according to the semi-quantitative workflow described in “Methods”. Following data processing (parameters provided in Additional file 1: Table S4), 65 peaks were retained, out of which 50 were identified compounds and 15 were unknowns. The identified metabolites comprised different classes of low molecular weight compounds including amino acids, organic acids and fatty acids as shown in Table 3. The full list of retained peaks, identified and unknown, used for model construction is provided in Additional file 1: Table S5.

**Table 3:** Metabolites identified by GC/MS

Amino acids, intermediates and derivatives		
Alanine	Glycine	Serine
Asparagine	Isoleucine	Threonine
Aspartic acid	Leucine	Tryptophan
Cystathionine	Lysine	Tyrosine
Cystine	Phenylalanine	Valine
Glutamic acid	Proline	
Glutamine	Pyroglutamic acid	
Glycolysis and TCA cycle compounds		
Isocitric acid/citric acid	Malic acid	Oxalacetic acid/pyruvic acid
Nucleic acids and intermediates		
Orotic acid	Uracil	
Urea cycle compounds		
Ornithine	Urea	
Polyamines		
Cadaverine	Spermidine	
Others		
2-Aminoadipic acid	Lactic acid	Plamitic acid (16:0)
4-Aminobenzoic acid	*n*-Propylamine	Quinolinic acid
Glucarate	Phthalic acid	Stearic acid (17:0)

Two compounds separated by a slash (/) indicates that the compounds could not be differentiated by our analytical method. For more information see also Additional file 1: Table S5

### Regression modeling by means of Orthogonal Projections to Latent Structures (OPLS)

Many methods exist in the literature for the mining and interpretation of metabolomics data. For regression modeling of a response variable taking continuous values, Orthogonal Projections to Latent Structures (OPLS) [32] is a well-known and widely employed method. Briefly, OPLS reduces high-dimensional predictor data into a small number of latent variables (or ‘components’) describing the variation in the predictors. One of the components (the predictive component) will be maximally correlated with the response, while other components (the orthogonal components) will describe predictor variation that is uncorrelated with the response. This partitioning of variance allows the removal of systematic error (which in the case of metabolomics data may easily arise from systematic or random error at various steps of the sample preparation or analysis) that may interfere with the regression of the response variable, hence the method’s suitability to metabolomics data analysis. OPLS has been successfully applied to the prediction of green tea ranking [30], developmental stage [21], and yeast replicative lifespan [22]. In this study, we also decided to employ this method for the regression modeling of growth rates under 1-butanol stress; here, metabolite peak intensity values were used as the predictor variables (*x*-variables), while growth rate under stress was used as the response variable (*y*-variable).

**Fig. 4:**
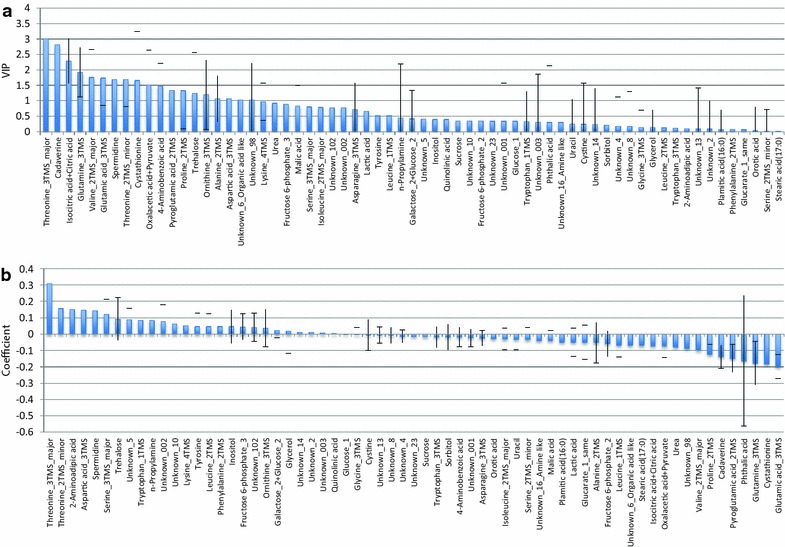
Metabolite scores according to the OPLS model. **a** VIP scores. **b** Coefficients. See text for a detailed explanation of these scores. The *error bars* in the VIP and coefficient plots represent standard error estimated from cross-validation

An OPLS model with 4 components (1 predictive, 3 orthogonal) was constructed from 75 samples and 65 predictor variables. The model described the response variable well (*R*^2^*Y* = 0.835) and was relatively robust (*Q*^2^ = 0.554, which was considered sufficient for a biological model [33]). The growth rate values predicted by the model also closely matched the actual measurements (Fig. 3). To identify metabolites correlated with growth rate under 1-butanol stress, we examined the Variable Importance in the Projection (VIP) and the PLS coefficient (Coeff) for each metabolite. In the case of a single-response OPLS model, the VIP (Fig. 4a) of a predictor (metabolite) simply indicates the absolute value of the correlation coefficient between that predictor and the response variable (growth rate). On the other hand, the Coeff (Fig. 4b) expresses the correlation between the predictor and response after the addition of orthogonal components. Each successive orthogonal component captures some variation in the predictor data, which is then subtracted from the data matrix, leaving a residual matrix which may show different correlation patterns from the original dataset. Hence, the Coeff plot may capture additional correlations between predictor and response variables in the residual dataset. The Coeff plot also preserves the sign of the correlations (positive or negative), while the VIP plot does not provide this information. Hence, the Coeff plot is useful for determining whether a predictor is positively or negatively correlated with response, and suggests whether a metabolite should be increased or decreased in order to increase the stress growth rate.

As shown in Fig. 4a, metabolite peaks with high VIP scores were (in order of decreasing VIP): Threonine_3TMS_major, Cadavarine, Isocitric acid + citric acid, Glutamine_3TMS, Valine_2TMS_major, Glutamic acid_3TMS, Threonine_2TMS_minor (Spermidine was omitted because of its large error bar). On the other hand, peaks with large values of Coeff (Fig. 4b) were: Threonine_3TMS_major, Threonine_2TMS_minor, 2-Aminoadipic acid, Aspartic acid_3TMS (positive Coeff); Glutamic acid_3TMS and Cystathionine (negative Coeff). The peaks listed above were considered on the basis of not only their VIP or Coeff values but also the size of the error bars in the plots, which indicate standard error values calculated from seven jackknife subsets of the data under the cross-validation procedure. In some cases multiple peaks may correspond to the same compound, e.g., two peaks (derivatives with 2 and 3 TMS groups, respectively) resulted from threonine. By combining the information from VIP and Coeff plots, a list of potentially important metabolites was made (Table 4).

**Table 4:** Potential 1-butanol tolerance-related metabolites identified from the OPLS model

Metabolite	High VIP	Large coeff	Correlation
2-Aminoadipic acid		✔	Positive
*Aspartic acid*		✔	*Positive*
Cadavarine	✔		Negative
Cystathionine		✔	Negative
*Glutamic acid*	✔	✔	*Negative*
*Glutamine*	✔		*Negative*
*Isocitric acid* *+* *citric acid*	✔		*Negative*
*Threonine*	✔	✔	*Positive*
Valine	✔		Negative

Italics indicates that the metabolite was subsequently considered for validation using metabolic enzyme deletion mutant(s)

### Selection of new 1-butanol tolerant strains based on OPLS model results

**Fig. 5:**
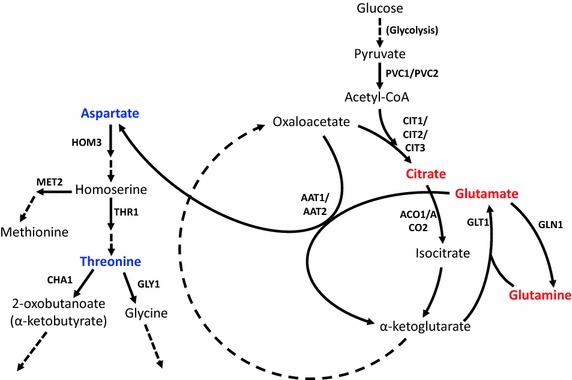
Metabolic pathways and genes related to threonine biosynthesis and the TCA cycle. Metabolites marked in *bold blue* (threonine, aspartate) were positively correlated with tolerance, while metabolites marked in *bold red* (citrate, glutamate, glutamine) were negatively correlated with tolerance

From the OPLS model, a list of metabolites correlated with, and potentially interacting with 1-butanol stress growth rate was identified (Table 4). The location of each metabolite in the yeast metabolic pathway network was looked up in the Yeast Biochemical Pathway Database (YeastCyc), a readily available online resource [34]. Out of these metabolites, we decided to focus on the part of the metabolic network centered on the TCA cycle and involving threonine biosynthesis from aspartate (see Fig. 5). The threonine–glycine biosynthetic pathway is predicted to be part of a so-called high-flux backbone of metabolism, containing sufficient flux for providing growth homeostasis in response to growth limiting perturbations [35]. Furthermore, this pathway has been shown to be involved in buffering growth inhibition by hydroxyurea in *S. cerevisiae* [36, 37]. Hence, it is possible that this pathway may also buffer growth inhibition due to 1-butanol, which would explain the observed correlation between threonine and glycine levels and the growth rate under 1-butanol stress. On the other hand, the TCA cycle is a major hub in cellular metabolism, playing a central role in energy production as well as providing precursors for the biosynthesis of a wide range of metabolites [38]. The close proximity of the TCA cycle and threonine biosynthesis, as well as the fact that the reactions driving these pathways as well as the genes governing them are well characterized, were major factors in our consideration.

It is important to note that the metabolites in Table 4 were determined on the basis of correlation, and a causal relationship with the growth rate under 1-butanol stress cannot be established using the OPLS model alone. On the other hand, it has been shown that by targeting the metabolites correlated with an objective phenotype, gene targets for modifying the phenotype could be selected [22]. Hence, we decided to test the hypothesis that some of these metabolites have a direct causal link with growth rate under stress.

Since threonine appeared at the top of both VIP and Coeff plots (Fig. 4), we focused on ways to increase threonine through gene deletion. Two potential deletions were identified: *cha1∆*, expected to reduce threonine flow to 2-oxobutanoate and increase threonine accumulation, and *met2∆*, which was postulated to increase threonine biosynthesis by reducing flux from homoserine into methionine biosynthesis and focusing the flux from aspartic acid towards threonine production (Fig. 5). On the other hand, several metabolites at or near the entry into TCA cycle were negatively correlated with growth rate under stress. We postulated that decreasing the carbon flow from glycolysis into the TCA cycle may affect the growth rate. Therefore, we proposed the deletion of various genes coding for isoforms of citrate synthase—*cit1*∆, *cit2*∆, *cit3*∆. As an additional step, we also investigated whether *aat1*∆ or *aat2*∆ would affect growth rate under stress by reducing carbon flow from oxaloacetate to aspartate (and ultimately threonine) and decreasing glutamate consumption (increasing its accumulation).

In this way, the OPLS regression model built upon metabolome data was used to determine potential gene targets for increasing the growth rate under 1-butanol stress of *S. cerevisiae*. The proposed strains are as summarized in Table 5.

**Table 5:** New strains selected according to important metabolites in the model

Strain	Enzyme encoded by gene	Rationale for selection
*cha1*∆	l-serine/l-threonine deaminase	Reducing threonine conversion into 2-oxobutanoate may increase threonine accumulation
*met2*∆	l-homoserine-*O*-acetyltransferase	Preventing carbon flow from homoserine into methionine biosynthesis may increase threonine production
*cit1*∆	Citrate synthase	Reducing acetyl-CoA and oxaloacetate condensation may decrease citrate level and/or TCA cycle activity
*cit2*∆	Citrate synthase	Reducing acetyl-CoA and oxaloacetate condensation may decrease citrate level and/or TCA cycle activity
*cit3*∆	Citrate synthase	Reducing acetyl-CoA and oxaloacetate condensation may decrease citrate level and/or TCA cycle activity
*aat1*∆	Aspartate aminotransferase	Reducing transamination of oxaloacetate and glutamate to aspartate and α-ketoglutarate may decrease threonine production and increase glutamate accumulation
*aat2*∆	Aspartate aminotransferase	Reducing transamination of oxaloacetate and glutamate to aspartate and α-ketoglutarate may decrease threonine production and increase glutamate accumulation

### Tolerance measurement and metabolome analysis of new strains

The proposed strains were then obtained from the EUROSCARF collection for validation of our strategy. In addition, since all the strains studied so far were deletion mutants constructed by gene disruption via KanMX cassette insertion (see “Methods”), we decided to obtain a similarly constructed mutant from the same deletion collection (EUROSCARF) to use as a control strain. We chose *his3*∆, since the *HIS3* gene is already nonfunctional in the parental strain BY4742, and insertion of a KanMX marker at this gene locus should not produce any effect due to loss of the gene’s function [14].

**Fig. 6:**
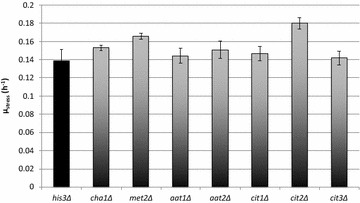
Experimental results for new strains selected based on model. Specific growth rates, under 1.5 % (v/v) 1-butanol stress condition, for new strains selected based on the model. *Columns* represent average values and *error bars* represent standard deviations from at least 3 replicate measurements. The measurement values are found in Additional file 1: Table S6

The new strains were subjected to tolerance measurement using the same procedure and stress condition (1.5 % (v/v) 1-butanol) as before. The results are as shown in Fig. 6. Among the seven new strains predicted to have perturbed metabolite levels, three strains indeed displayed higher growth rate under stress relative to the control strain *his3*∆. Furthermore, these increases were statistically significant (*p* values <0.1) according to Student’s *t* test (one-tailed, unequal variance) (see Additional file 1: Table S6).

**Fig. 7:**
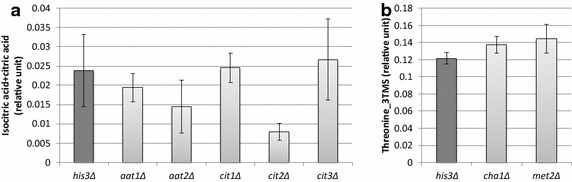
Experimental results for new strains selected based on model. **a** Relative threonine levels for *cha1*∆, *met2*∆, and *his3*∆ (control strain). *Columns* represent average values from *n* = 4 samples. **b** Relative citrate levels for *cit1*-*3*∆, *aat1*-*2*∆, and *his3*∆. *Columns* represent average values from *n* = 4 samples

Two of the strains exhibiting higher growth rate under stress, *met2*∆ and *cha1*∆, were selected on the basis of increasing threonine. By subjecting these strains to metabolome analysis, we confirmed that these two strains indeed had higher threonine relative to the control (Fig. 7a). This shows that the correlation between threonine and stress growth rate still held true for new strains selected from outside the set of strains originally used to construct the model (the ‘training set’). On the other hand, out of five TCA cycle related strains, only *cit2*∆ displayed higher growth rate under stress compared to the control strain. However, this strain was also the only one to show a significant decrease in citrate relative to control (Fig. 7b). From this we can observe that (1) only *cit2*∆ is active and involved in regulating citric acid level in yeast under the investigated condition, and (2) the correlation between citric acid and stress growth rate again holds true even for new strains selected from outside the training set.

From the above, it was shown that our model successfully described a relationship between metabolome and growth rate under 1-butanol stress that was also observed in the new selected strains. This suggests that the increased growth rates were due to the metabolite changes effected by gene deletions determined according to the model predictions. Thus, it could be concluded that the identification of gene targets based on comparison of metabolome data from multiple strains was a valid, useful strategy for phenotype improvement.

### Considerations for future developments

As a primarily data-driven approach, successful application of the semi-rational strategy depends in large part on the type, quality and quantity of data available. Hence, results may be greatly influenced by the strains used as well as choice of analytical platform. The choice of putative transcription factor mutants to obtain variety of metabolic profiles and growth rate phenotypes seemed appropriate here; an alternative could be using a set of metabolic enzyme deletion mutants to simplify interpretation of the cause-and-effect relationship between gene and metabolites. GC/MS was chosen as the analytical platform for its high reproducibility and good separation, as described above; however, other platforms that cover different portions of the metabolome, for example LC/MS or CE/MS are also attractive as alternative or complementary methods that could be considered in future implementations of this strategy.

In this study, metabolome data sampled under stress-free condition was used for the prediction of growth rate phenotype under stress condition. This suggests that a dataset of metabolite profiles from a large collection of strains sampled under a standard, stress-free condition could be useful for the prediction of multiple phenotypes and subsequent identification of metabolites or mechanisms contributing to their improvement. We predict one difficulty in implementing such a strategy: it is possible that different subgroups of the data will show different, sometimes conflicting patterns of metabolite–phenotype relationship, and including the metabolome data from a large number of widely divergent strains may yield a poor-performing model that fail to indicate any significant metabolite. Therefore, the question of how to choose an optimal, informative set of samples for regression modeling will need to be addressed.

## Conclusions

In this study, a metabolomics-based strategy was proposed and applied, whereby strains were subjected to phenotype measurement and metabolome analysis, and a regression model for the target phenotype was constructed from metabolite data. Based on important metabolites indicated by the model, gene targets for increasing growth rate under 1-butanol stress were determined, and new strains with higher growth rates were predicted and validated. The results show the usefulness of metabolomics in semi-rational identification of between-strain differences that may be utilized in phenotype improvement.

The strategy adopted in this work represents a promising result in applied metabolomics research. Although metabolomics is a relatively new field with many remaining avenues for improvement—such as automation for increased throughput, or further technological advancements to increase coverage, specificity and reproducibility; and can be demanding in its technical requirements—for example, appropriate choice of platform and optimization of various parameters—we have shown that metabolomics in its current state can be useful as a standalone tool to identify and suggest gene targets for phenotype improvement. We expect that future studies applying this strategy to other microorganisms and/or phenotypes will establish it as a widely applicable methodology in strain engineering.

## Methods

### Strains and growth media

*Saccharomyces cerevisiae* single gene disruption mutants constructed on the BY4742 parental strain genetic background (*MATα leu2∆0 lys2∆0 ura3∆0 his3∆1*) were used in this work. The mutant strains were from the EUROSCARF collection and constructed by replacing the target genes with *KanMX* cassettes according to the strategy used in the Saccharomyces Genome Deletion Project [39].

YPD agar medium [10 g Bacto™ yeast extract (BD, NJ, USA), 20 g Bacto™ peptone (BD, NJ, USA), 20 g d-glucose (Nacalai Tesque, Kyoto, Japan) and 20 g agar (Nacalai Tesque, Kyoto, Japan) per 1 L medium] was used for propagation of strains, while yeast synthetic complete (SC) medium with defined composition [6.7 g Difco™ yeast nitrogen base without amino acids (BD, NJ, USA), 1.92 g yeast synthetic drop-out media supplement without uracil (Sigma-Aldrich, MO, USA), 76 mg uracil (Sigma-Aldrich, MO, USA) and 20 g d-glucose dissolved in distilled water and filter-sterilized, then added to separately autoclaved distilled water to yield 1 L total medium] was used for all liquid cultures for the metabolomics and tolerance measurements.

### Measurement of 1-butanol tolerance

*Saccharomyces cerevisiae* gene disruption strains carrying the kanamycin resistance marker were streaked from −80 °C deep freeze storage onto YPD agar plates supplemented with 250 µg/mL G418 antibiotic (Sigma-Aldrich, MO, USA) and incubated at 30 °C until single colonies are sufficiently large to be isolated and transferred to a fresh YPD agar plate. The plates were incubated overnight then kept at 4 °C to be used as working stock.

All cultivation steps were conducted in 50-mL Falcon centrifuge tubes (BD, NJ, USA) capped with gas-permeable silicon plugs (Shin-Etsu Polymer, Tokyo, Japan) at 30 °C with rotational shaking on a Bio-Shaker BR-40LF (Taitec, Tokyo, Japan) at 200 rpm. Pre-precultures of each strain were prepared by inoculating 3 mL SC medium with cells from working stock. After 9–10 h, pre-precultures were diluted into 7 mL SC medium so that starting OD_600_ = 0.01 and precultured for a further 13 h. For the main cultures, precultures were diluted into 6 mL SC medium, spiked with 1.5 % (v/v) 1-butanol for the stress condition, so that initial OD_600_ = 0.15 and then cultivated as before. At 1 h (non-stress) or 2 h (stress condition) intervals, 200 µL of culture broth was taken from each sample tube for measurement of optical density at 600 nm (OD_600_) using an iMark microplate reader (Bio-Rad, CA, USA). Specific growth rates were calculated by regression of the blank-subtracted OD_600_ measurements against time using a minimum of 4 time-points.

### Sample preparation for metabolome analysis

Cultivation steps were performed identically to the tolerance measurement procedure described above. Sample collection, extraction and derivatization steps were performed as previously reported [40] with appropriate modifications. Main cultures were started at OD_600_ = 0.15 under non-stress condition, and OD_600_ was checked at appropriate intervals to determine the collection time. At OD_600_ = 2, which corresponds to the mid-exponential phase of cell growth for all the strains tested, cells were collected by transferring 5 mL of culture broth to a glass funnel from which culture medium was removed by vacuum suction through a 25 mm diameter, 0.45 µm pore size nylon membrane filter (Millipore, MA, USA). The filter-bound cells were then transferred into a 2-mL microcentrifuge tube (Eppendorf, Hamburg, Germany) containing 1 mL of extraction solvent (methanol:water:chloroform in 5:2:2 ratio; methanol and chloroform from Chameleon Reagent, Osaka, Japan; distilled water from Wako, Osaka, Japan) with 1.2 µg/mL ribitol (Wako, Osaka, Japan) added as internal standard, and the microtube was then rapidly cooled in liquid nitrogen to quench metabolism. The time for the collection process was kept within 30 s. Samples were then stored at −80 °C until extraction.

Metabolite extraction from the cells was performed by incubation at 4 °C with vigorous shaking (1200 rpm) on a Thermomixer Comfort (Eppendorf, Hamburg, Germany) for 30 min. 850 µL of solvent containing extracted metabolites was then taken from each tube and mixed with 500 µL of ultrapure water (Wako, Osaka, Japan), vortexed briefly then ultracentrifuged at 16,000 r.c.f., 4 °C for 3 min to separate the polar and nonpolar phases. 175 µL of the upper polar phase containing hydrophilic metabolites was further transferred to new 1.5-mL microcentrifuge tubes (Watson, NJ, USA) and methanol was removed from the samples by centrifugal concentration for 2 h using a VC-96R Spin Dryer Standard (Taitec, Tokyo, Japan) before overnight lyophilization in a VD-800F Freeze Dryer (Taitec, Tokyo, Japan) and storage at −30 °C.

Extracted metabolites were derivatized by oximation and silylation. The oximation reagent, methoxyamine hydrochloride (Sigma-Aldrich, MO, USA) was first dissolved in pyridine (Wako, Osaka, Japan) to a concentration of 10 mg/mL and 40 µL added to each sample tube containing the lyophilized extracts. After reaction at 30 °C, 1200 rpm for 90 min, 50 µL of *N*-methyl-*N*-(trimethylsilyl)trifluoroacetamide (MSTFA) (GL Sciences, Tokyo, Japan) was added and the silylation reaction was performed at 37 °C, 1200 rpm for 30 min. The derivatized samples were transferred to glass vials (Chromacol, Hertfordshire, UK) and analyzed within 24 h.

### GC/MS analysis

Instrumental analysis was performed on a GCMS-QP2010 Ultra (Shimadzu, Kyoto, Japan) gas chromatograph coupled with quadrupole mass spectrometer equipped with an AOC-20i/s autoinjector (Shimadzu, Kyoto, Japan). An InertCap 5MS/NP column (GL Sciences, Tokyo, Japan) 0.25 mm ID × 30 m, df = 0.25 μm was used for the GC separation. The mass spectrometer was auto-tuned and calibrated prior to analysis. 1 μL of sample was injected in split mode with a split ratio of 1:25. The inlet temperature was set at 230 °C and column flow rate was 1.12 mL/min (linear velocity 39 cm/s). The column temperature was held at 80 °C for 2 min, raised by 15 °C/min to 330 °C, and held at 330 °C for 6 min. The transfer line and ion source temperatures were 250 and 200 °C, respectively. Electron ionization (EI) was performed at 70 eV. The mass range of the detector was set to *m*/*z* 85–500 and the detector voltage was set by auto-tuning to a value between 1.00 and 2.00 kV.

An alkane standard mix was prepared from 1:1:1 mix of pyridine, C8–C20 and C21–C40 alkane standard solutions, and injected at the start of each analytical run for calculating retention indices. In addition, a blank pyridine sample was injected every six samples for diagnostic purposes (to check for column bleed and carryover).

### Data processing

GC/MS raw data files were converted into netCDF (*.cdf) format according to the ANDI (Analytical Data Interchange Protocol) specification using the proprietary software GCMSsolution (Shimadzu, Kyoto, Japan) before peak detection, baseline correction and retention time alignment using the freely available data processing tool MetAlign [41]. MSClust [42] was used to assign putative compound memberships in an unsupervised manner to each mass peak, and the peak retention times were adjusted according to compound membership. The adjusted data matrices were then imported into AIoutput2 ver.1.29 [31] for automated RI-based compound identification and quantification. The parameters used for MetAlign, MSClust and AIoutput2 are provided in Additional file 1: Table S4. To account for deviations in overall intensity due to volumetric errors in sample collection and preparation as well as systematic drift or random fluctuations in ionization efficiency and mass detector sensitivity, peak intensities of each sample were normalized to the internal standard (ribitol) peak.

### Multivariate analysis

Multivariate analysis was performed using SIMCA-P+ version 12 (Umterics, Umeå, Sweden). Regression models were constructed by means of Orthogonal Projections to Latent Structures (OPLS), using unit variance (UV)-scaled metabolite intensities as predictor variables and specific growth rates under 1-butanol stress as response variables.

## Additional file

**Additional file 1:** Supplemental tables. Full data for various tables and charts found in the text.

## Abbreviations

GC/MS: gas chromatography coupled to mass spectrometry
OPLS: orthogonal projections to latent structures
VIP: variable importance in the projection
RMSEE: root mean squared error of estimation
SC: synthetic complete
YPD: yeast extract peptone dextrose
