# Supplementary material for: A metabolomics-based strategy for identification of gene targets for phenotype improvement and its application to 1-butanol tolerance in Saccharomyces cerevisiae
Source: Biotechnol Biofuels. 2015 Sep 15;8:144. doi: 10.1186/s13068-015-0330-z (PMC4570087; doi:10.1186/s13068-015-0330-z)
Supplement: Supplementary file 1 — Additional file 1: Supplemental tables. Full data for various tables and charts found in the text. [file 13068_2015_330_MOESM1_ESM.docx]

**Supplementary material**

**Table S1. Strains used in this study**

| Strain | I | II | Description in Saccharomyces Genome Database |
| --- | --- | --- | --- |
| *ace1/cup2* | 1 |  | Copper-binding transcription factor; activates transcription of the metallothionein genes CUP1-1 and CUP1-2 in response to elevated copper concentrations |
| *ace2* |  |  | Transcription factor required for septum destruction after cytokinesis; phosphorylation by Cbk1p blocks nuclear exit of Ace2p during the M-to-G1 transition, causing its specific localization to daughter cell nuclei, and also increases Ace2p activity; phosphorylation by Cdc28p and Pho85p prevents nuclear import during cell cycle phases other than cytokinesis; part of the RAM network that regulates cellular polarity and morphogenesis |
| *ada2* |  |  | Transcription coactivator, component of the ADA and SAGA transcriptional adaptor/HAT (histone acetyltransferase) complexes |
| *adr1* |  |  | Carbon source-responsive zinc-finger transcription factor, required for transcription of the glucose-repressed gene ADH2, of peroxisomal protein genes, and of genes required for ethanol, glycerol, and fatty acid utilization |
| *aft1* | 1 |  | Transcription factor involved in iron utilization and homeostasis; binds the consensus site PyPuCACCCPu and activates the expression of target genes in response to changes in iron availability; in iron-replete conditions activity is negatively regulated by Grx3p, Grx4p, and Fra2p, which regulate Aft1p translocation from the nucleus to the cytoplasm |
| *aft2* | 1 |  | Iron-regulated transcriptional activator; activates genes involved in intracellular iron use and required for iron homeostasis and resistance to oxidative stress; similar to Aft1p |
| *arg80* |  |  | Transcription factor involved in regulation of arginine-responsive genes; acts with Arg81p and Arg82p |
| *arg81/argr2* |  |  | Zinc-finger transcription factor of the Zn(2)-Cys(6) binuclear cluster domain type, involved in the regulation of arginine-responsive genes; acts with Arg80p and Arg82p |
| *arg82* |  |  | Inositol polyphosphate multikinase (IPMK), sequentially phosphorylates Ins(1,4,5)P3 to form Ins(1,3,4,5,6)P5; also has diphosphoinositol polyphosphate synthase activity; regulates arginine-, phosphate-, and nitrogen-responsive genes |
| *aro80* |  | 1 | Zinc finger transcriptional activator of the Zn2Cys6 family; activates transcription of aromatic amino acid catabolic genes in the presence of aromatic amino acids |
| *arr1* | 1 |  | Transcriptional activator of the basic leucine zipper (bZIP) family, required for transcription of genes involved in resistance to arsenic compounds |
| *arr1* | 1 |  | Transcriptional activator of the basic leucine zipper (bZIP) family, required for transcription of genes involved in resistance to arsenic compounds |
| *azf1* | 1 |  | Zinc-finger transcription factor, involved in induction of CLN3 transcription in response to glucose; genetic and physical interactions indicate a possible role in mitochondrial transcription or genome maintenance |
| *bas1* |  | 1 | Myb-related transcription factor involved in regulating basal and induced expression of genes of the purine and histidine biosynthesis pathways; also involved in regulation of meiotic recombination at specific genes |
| *bas2/grf10/pho2* |  | 1 | Homeobox transcription factor; regulatory targets include genes involved in phosphate metabolism; binds cooperatively with Pho4p to the PHO5 promoter; phosphorylation of Pho2p facilitates interaction with Pho4p |
| *cad1/yap2* | 1 | 1 | AP-1-like basic leucine zipper (bZIP) transcriptional activator involved in stress responses, iron metabolism, and pleiotropic drug resistance; controls a set of genes involved in stabilizing proteins; binds consensus sequence TTACTAA |
| *cat8* |  |  | Zinc cluster transcriptional activator necessary for derepression of a variety of genes under non-fermentative growth conditions, active after diauxic shift, binds carbon source responsive elements |
| *cbf1* |  |  | Dual function helix-loop-helix protein; binds the motif CACRTG present at several sites including MET gene promoters and centromere DNA element I (CDEI); affects nucleosome positioning at this motif; associates with other transcription factors such as Met4p and Isw1p to mediate transcriptional activation or repression; associates with kinetochore proteins and required for efficient chromosome segregation |
| *cha4* |  | 1 | DNA binding transcriptional activator, mediates serine/threonine activation of the catabolic L-serine (L-threonine) deaminase (CHA1); Zinc-finger protein with Zn[2]-Cys[6] fungal-type binuclear cluster domain |
| *cin5* | 1 |  | Basic leucine zipper (bZIP) transcription factor of the yAP-1 family; physically interacts with the Tup1-Cyc8 complex and recruits Tup1p to its targets; mediates pleiotropic drug resistance and salt tolerance; nuclearly localized under oxidative stress and sequestered in the cytoplasm by Lot6p under reducing conditions |
| *crz1* | 1 |  | Transcription factor that activates transcription of genes involved in stress response; nuclear localization is positively regulated by calcineurin-mediated dephosphorylation |
| *cst6* | 1 |  | Basic leucine zipper (bZIP) transcription factor, in ATF/CREB family; mediates transcriptional activation of NCE103 (encoding carbonic anhydrase) in response to low CO2 levels such as in the ambient air; proposed to be a regulator of oleate responsive genes; involved in utilization of non-optimal carbon sources and chromosome stability |
| *cup9* |  | 1 | Homeodomain-containing transcriptional repressor of PTR2, which encodes a major peptide transporter; imported peptides activate ubiquitin-dependent proteolysis, resulting in degradation of Cup9p and de-repression of PTR2 transcription |
| *dal80* |  |  | Negative regulator of genes in multiple nitrogen degradation pathways; expression is regulated by nitrogen levels and by Gln3p; member of the GATA-binding family, forms homodimers and heterodimers with Deh1p |
| *dal81* |  | 1 | Positive regulator of genes in multiple nitrogen degradation pathways; contains DNA binding domain but does not appear to bind the dodecanucleotide sequence present in the promoter region of many genes involved in allantoin catabolism |
| *dal82* |  |  | Positive regulator of allophanate inducible genes; binds a dodecanucleotide sequence upstream of all genes that are induced by allophanate; contains an UISALL DNA-binding, a transcriptional activation, and a coiled-coil domain |
| *ecm22* |  | 1 | Sterol regulatory element binding protein, regulates transcription of sterol biosynthetic genes; contains Zn[2]-Cys[6] binuclear cluster; homologous to Upc2p; relocates from intracellular membranes to perinuclear foci on sterol depletion |
| *fzf1* |  | 1 | Transcription factor involved in sulfite metabolism, sole identified regulatory target is SSU1, overexpression suppresses sulfite-sensitivity of many unrelated mutants due to hyperactivation of SSU1, contains five zinc fingers |
| *gal3* | 1 |  | Transcriptional regulator involved in activation of the GAL genes in response to galactose; forms a complex with Gal80p to relieve Gal80p inhibition of Gal4p; binds galactose and ATP but does not have galactokinase activity |
| *gal4* | 1 |  | DNA-binding transcription factor required for the activation of the GAL genes in response to galactose; repressed by Gal80p and activated by Gal3p |
| *gal80* |  |  | Transcriptional regulator involved in the repression of GAL genes in the absence of galactose; inhibits transcriptional activation by Gal4p; inhibition relieved by Gal3p or Gal1p binding |
| *gat1* |  | 1 | Transcriptional activator of genes involved in nitrogen catabolite repression; contains a GATA-1-type zinc finger DNA-binding motif; activity and localization regulated by nitrogen limitation and Ure2p |
| *gat2* |  |  | Protein containing GATA family zinc finger motifs; similar to Gln3p and Dal80p; expression repressed by leucine |
| *gcn4* | 1 | 1 | Basic leucine zipper (bZIP) transcriptional activator of amino acid biosynthetic genes in response to amino acid starvation; expression is tightly regulated at both the transcriptional and translational levels |
| *gcr2* |  |  | Transcriptional activator of genes involved in glycolysis; interacts and functions with the DNA-binding protein Gcr1p |
| *gis1* |  |  | JmjC domain-containing histone demethylase and transcription factor; involved in expression of genes during nutrient limitation; negatively regulates DPP1 and PHR1; activity is modulated by limited proteasome-mediated proteolysis; has a JmjC and a JmjN domain in the N-terminal region that interact, promoting Gis1p stability and proper transcriptional activity; contains transactivating domains TAD1 and TAD2 downstream of the Jmj domains and a C-terminal DNA binding domain |
| *gln3* |  | 1 | Transcriptional activator of genes regulated by nitrogen catabolite repression (NCR), localization and activity regulated by quality of nitrogen source |
| *gzf3* |  | 1 | GATA zinc finger protein and Dal80p homolog that negatively regulates nitrogen catabolic gene expression by competing with Gat1p for GATA site binding; function requires a repressive carbon source; dimerizes with Dal80p and binds to Tor1p |
| *hac1* | 1 | 1 | Basic leucine zipper (bZIP) transcription factor (ATF/CREB1 homolog) that regulates the unfolded protein response, via UPRE binding, and membrane biogenesis; ER stress-induced splicing pathway facilitates efficient Hac1p synthesis |
| *hal9* | 1 |  | Transcription factor that activates transcription of genes involved in stress response; nuclear localization is positively regulated by calcineurin-mediated dephosphorylation |
| *hap2* |  |  | Subunit of the heme-activated, glucose-repressed Hap2p/3p/4p/5p CCAAT-binding complex, a transcriptional activator and global regulator of respiratory gene expression; contains sequences sufficient for both complex assembly and DNA binding |
| *hap3* |  |  | Subunit of the heme-activated, glucose-repressed Hap2p/3p/4p/5p CCAAT-binding complex, a transcriptional activator and global regulator of respiratory gene expression; contains sequences contributing to both complex assembly and DNA binding |
| *hap4* |  |  | Subunit of the heme-activated, glucose-repressed Hap2p/3p/4p/5p CCAAT-binding complex, a transcriptional activator and global regulator of respiratory gene expression; provides the principal activation function of the complex |
| *hap5* |  |  | Subunit of the heme-activated, glucose-repressed Hap2/3/4/5 CCAAT-binding complex, a transcriptional activator and global regulator of respiratory gene expression; required for assembly and DNA binding activity of the complex |
| *ime1* |  |  | Master regulator of meiosis that is active only during meiotic events, activates transcription of early meiotic genes through interaction with Ume6p, degraded by the 26S proteasome following phosphorylation by Ime2p |
| *ino2* | 1 | 1 | Component of the heteromeric Ino2p/Ino4p basic helix-loop-helix transcription activator that binds inositol/choline-responsive elements (ICREs), required for derepression of phospholipid biosynthetic genes in response to inositol depletion |
| *ino4* |  | 1 | Transcription factor required for derepression of inositol-choline-regulated genes involved in phospholipid synthesis; forms a complex, with Ino2p, that binds the inositol-choline-responsive element through a basic helix-loop-helix domain |
| *ixr1* |  |  | Protein that binds DNA containing intrastrand cross-links formed by cisplatin, contains two HMG (high mobility group box) domains, which confer the ability to bend cisplatin-modified DNA; mediates aerobic transcriptional repression of COX5b |
| *leu3* |  | 1 | Zinc-knuckle transcription factor, repressor and activator; regulates genes involved in branched chain amino acid biosynthesis and ammonia assimilation; acts as a repressor in leucine-replete conditions and as an activator in the presence of alpha-isopropylmalate, an intermediate in leucine biosynthesis that accumulates during leucine starvation |
| *lys14* |  | 1 | Transcriptional activator involved in regulation of genes of the lysine biosynthesis pathway; requires 2-aminoadipate semialdehyde as co-inducer |
| *mac1* |  | 1 | Copper-sensing transcription factor involved in regulation of genes required for high affinity copper transport |
| *met28* |  | 1 | Basic leucine zipper (bZIP) transcriptional activator in the Cbf1p-Met4p-Met28p complex, participates in the regulation of sulfur metabolism |
| *met31* |  | 1 | Zinc-finger DNA-binding protein, involved in transcriptional regulation of the methionine biosynthetic genes, similar to Met32p |
| *mga1* |  |  | Protein similar to heat shock transcription factor; multicopy suppressor of pseudohyphal growth defects of ammonium permease mutants |
| *mig1* |  |  | Transcription factor involved in glucose repression; sequence specific DNA binding protein containing two Cys2His2 zinc finger motifs; regulated by the SNF1 kinase and the GLC7 phosphatase |
| *mig2* |  |  | Protein containing zinc fingers, involved in repression, along with Mig1p, of SUC2 (invertase) expression by high levels of glucose; binds to Mig1p-binding sites in SUC2 promoter |
| *mig3* | 1 |  | Probable transcriptional repressor involved in response to toxic agents such as hydroxyurea that inhibit ribonucleotide reductase; phosphorylation by Snf1p or the Mec1p pathway inactivates Mig3p, allowing induction of damage response genes |
| *mks1* |  | 1 | Pleiotropic negative transcriptional regulator involved in Ras-CAMP and lysine biosynthetic pathways and nitrogen regulation; involved in retrograde (RTG) mitochondria-to-nucleus signaling |
| *mot3* | 1 | 1 | Transcriptional repressor and activator with two C2-H2 zinc fingers; involved in repression of a subset of hypoxic genes by Rox1p, repression of several DAN/TIR genes during aerobic growth, and repression of ergosterol biosynthetic genes in response to hyperosmotic stress; contributes to recruitment of the Tup1p-Cyc8p general repressor to promoters; involved in positive transcriptional regulation of CWP2 and other genes; can form the [MOT3+] prion |
| *msn1* | 1 |  | Transcriptional activator involved in regulation of invertase and glucoamylase expression, invasive growth and pseudohyphal differentiation, iron uptake, chromium accumulation, and response to osmotic stress; localizes to the nucleus |
| *msn2* | 1 |  | Transcriptional activator related to Msn4p; activated in stress conditions, which results in translocation from the cytoplasm to the nucleus; binds DNA at stress response elements of responsive genes, inducing gene expression |
| *msn4* | 1 |  | Transcriptional activator related to Msn2p; activated in stress conditions, which results in translocation from the cytoplasm to the nucleus; binds DNA at stress response elements of responsive genes, inducing gene expression |
| *ngg1/ada3* |  |  | Transcriptional regulator involved in glucose repression of Gal4p-regulated genes; component of transcriptional adaptor and histone acetyltransferase complexes, the ADA complex, the SAGA complex, and the SLIK complex |
| *not3* |  |  | Subunit of the CCR4-NOT complex, which is a global transcriptional regulator with roles in transcription initiation and elongation and in mRNA degradation |
| *nrg1* | 1 |  | Transcriptional repressor that recruits the Cyc8p-Tup1p complex to promoters; mediates glucose repression and negatively regulates a variety of processes including filamentous growth and alkaline pH response |
| *oaf1* |  |  | Oleate-activated transcription factor, acts alone and as a heterodimer with Pip2p; activates genes involved in beta-oxidation of fatty acids and peroxisome organization and biogenesis |
| *opi1* |  | 1 | Transcriptional regulator of a variety of genes; phosphorylation by protein kinase A stimulates Opi1p function in negative regulation of phospholipid biosynthetic genes; involved in telomere maintenance |
| *pdr1* | 1 |  | Zinc cluster protein that is a master regulator involved in recruiting other zinc cluster proteins to pleiotropic drug response elements (PDREs) to fine tune the regulation of multidrug resistance genes |
| *pdr3* | 1 | 1 | Transcriptional activator of the pleiotropic drug resistance network, regulates expression of ATP-binding cassette (ABC) transporters through binding to cis-acting sites known as PDREs (PDR responsive elements); post-translationally up-regulated in cells lacking a functional mitochondrial genome |
| *pdr8* | 1 | 1 | Transcription factor; targets include ATP-binding cassette (ABC) transporters, major facilitator superfamily transporters, and other genes involved in the pleiotropic drug resistance (PDR) phenomenon |
| *pho23* |  |  | Probable component of the Rpd3 histone deacetylase complex, involved in transcriptional regulation of PHO5; affects termination of snoRNAs and cryptic unstable transcripts (CUTs); C-terminus has similarity to human candidate tumor suppressor p33(ING1) and its isoform ING3 |
| *pho4* | 1 |  | Basic helix-loop-helix (bHLH) transcription factor of the myc-family; activates transcription cooperatively with Pho2p in response to phosphate limitation; binding to 'CACGTG' motif is regulated by chromatin restriction, competitive binding of Cbf1p to the same DNA binding motif and cooperation with Pho2p,; function is regulated by phosphorylation at multiple sites and by phosphate availability |
| *ppr1* | 1 | 1 | Zinc finger transcription factor containing a Zn(2)-Cys(6) binuclear cluster domain, positively regulates transcription of URA1, URA3, URA4, and URA10, which are involved in de novo pyrimidine biosynthesis, in response to pyrimidine starvation; activity may be modulated by interaction with Tup1p |
| *put3* |  |  | Transcriptional activator of proline utilization genes, constitutively binds PUT1 and PUT2 promoter sequences as a dimer and undergoes a conformational change to form the active state; differentially phosphorylated in the presence of different nitogen sources; has a Zn(2)-Cys(6) binuclear cluster domain |
| *rgt1* | 1 | 1 | Glucose-responsive transcription factor that regulates expression of several glucose transporter (HXT) genes in response to glucose; binds to promoters and acts both as a transcriptional activator and repressor |
| *ric1* |  | 1 | Protein involved in retrograde transport to the cis-Golgi network; forms heterodimer with Rgp1p that acts as a GTP exchange factor for Ypt6p; involved in transcription of rRNA and ribosomal protein genes |
| *rim101* | 1 |  | Transcriptional repressor involved in response to pH and in cell wall construction; required for alkaline pH-stimulated haploid invasive growth and sporulation; activated by proteolytic processing; similar to A. nidulans PacC |
| *rox1* |  |  | Heme-dependent repressor of hypoxic genes; contains an HMG domain that is responsible for DNA bending activity |
| *rpn4* | 1 |  | Transcription factor that stimulates expression of proteasome genes; Rpn4p levels are in turn regulated by the 26S proteasome in a negative feedback control mechanism; RPN4 is transcriptionally regulated by various stress responses |
| *rsf2* |  |  | Zinc-finger protein involved in transcriptional control of both nuclear and mitochondrial genes, many of which specify products required for glycerol-based growth, respiration, and other functions |
| *sfl1* | 1 |  | Transcriptional repressor and activator; involved in repression of flocculation-related genes, and activation of stress responsive genes; negatively regulated by cAMP-dependent protein kinase A subunit Tpk2p |
| *sin3* |  |  | Component of the Sin3p-Rpd3p histone deacetylase complex, involved in transcriptional repression and activation of diverse processes, including mating-type switching and meiosis; involved in the maintenance of chromosomal integrity |
| *sip4* |  |  | C6 zinc cluster transcriptional activator that binds to the carbon source-responsive element (CSRE) of gluconeogenic genes; involved in the positive regulation of gluconeogenesis; regulated by Snf1p protein kinase; localized to the nucleus |
| *skn7* | 1 |  | Nuclear response regulator and transcription factor; physically interacts with the Tup1-Cyc8 complex and recruits Tup1p to its targets; part of a branched two-component signaling system; required for optimal induction of heat-shock genes in response to oxidative stress; involved in osmoregulation |
| *sko1* | 1 |  | Basic leucine zipper transcription factor of the ATF/CREB family; forms a complex with Tup1p and Cyc8p to both activate and repress transcription; cytosolic and nuclear protein involved in osmotic and oxidative stress responses |
| *spt23* |  |  | ER membrane protein involved in regulation of OLE1 transcription, acts with homolog Mga2p; inactive ER form dimerizes and one subunit is then activated by ubiquitin/proteasome-dependent processing followed by nuclear targeting |
| *stb5* | 1 |  | Transcription factor, involved in regulating multidrug resistance and oxidative stress response; forms a heterodimer with Pdr1p; contains a Zn(II)2Cys6 zinc finger domain that interacts with a pleiotropic drug resistance element in vitro |
| *stp1* | 1 |  | Transcription factor, undergoes proteolytic processing by SPS (Ssy1p-Ptr3p-Ssy5p)-sensor component Ssy5p in response to extracellular amino acids; activates transcription of amino acid permease genes and may have a role in tRNA processing |
| *stp2* | 1 |  | Transcription factor, activated by proteolytic processing in response to signals from the SPS sensor system for external amino acids; activates transcription of amino acid permease genes |
| *sut1* |  |  | Transcription factor of the Zn[II]2Cys6 family involved in sterol uptake; involved in induction of hypoxic gene expression |
| *swi6* | 1 |  | Transcription cofactor; forms complexes with Swi4p and Mbp1p to regulate transcription at the G1/S transition; involved in meiotic gene expression; also binds Stb1p to regulate transcription at START; cell wall stress induces phosphorylation by Mpk1p, which regulates Swi6p localization; required for the unfolded protein response, independently of its known transcriptional coactivators |
| *tea1* |  |  | Ty1 enhancer activator required for full levels of Ty enhancer-mediated transcription; C6 zinc cluster DNA-binding protein |
| *thi2* |  | 1 | Transcriptional activator of thiamine biosynthetic genes; interacts with regulatory factor Thi3p to control expression of thiamine biosynthetic genes with respect to thiamine availability; acts together with Pdc2p to respond to thiaminediphosphate demand, possibly as related to carbon source availability; zinc finger protein of the Zn(II)2Cys6 type |
| *tye7* |  |  | Serine-rich protein that contains a basic-helix-loop-helix (bHLH) DNA binding motif; binds E-boxes of glycolytic genes and contributes to their activation; may function as a transcriptional activator in Ty1-mediated gene expression |
| *uga3* |  |  | Transcriptional activator necessary for gamma-aminobutyrate (GABA)-dependent induction of GABA genes (such as UGA1, UGA2, UGA4); zinc-finger transcription factor of the Zn(2)-Cys(6) binuclear cluster domain type; localized to the nucleus |
| *ume6* | 1 | 1 | Key transcriptional regulator of early meiotic genes, binds URS1 upstream regulatory sequence, couples metabolic responses to nutritional cues with initiation and progression of meiosis, forms complex with Ime1p, and also with Sin3p-Rpd3p |
| *usv1* | 1 | 1 | Putative transcription factor containing a C2H2 zinc finger; mutation affects transcriptional regulation of genes involved in growth on non-fermentable carbon sources, response to salt stress and cell wall biosynthesis |
| *wtm2* | 1 |  | Transcriptional modulator involved in regulation of meiosis, silencing, and expression of RNR genes; involved in response to replication stress; contains WD repeats |
| *xbp1* | 1 |  | Transcriptional repressor that binds to promoter sequences of the cyclin genes, CYS3, and SMF2; expression is induced by stress or starvation during mitosis, and late in meiosis; member of the Swi4p/Mbp1p family; potential Cdc28p substrate |
| *yap1* | 1 |  | Basic leucine zipper (bZIP) transcription factor required for oxidative stress tolerance; activated by H2O2 through the multistep formation of disulfide bonds and transit from the cytoplasm to the nucleus; mediates resistance to cadmium |
| *yap3* |  |  | Basic leucine zipper (bZIP) transcription factor |
| *yap5* |  |  | Basic leucine zipper (bZIP) iron-sensing transcription factor |
| *yap6* | 1 | 1 | Basic leucine zipper (bZIP) transcription factor; physically interacts with the Tup1-Cyc8 complex and recruits Tup1p to its targets; overexpression increases sodium and lithium tolerance; computational analysis suggests a role in regulation of expression of genes involved in carbohydrate metabolism |
| *yap7* |  |  | Putative basic leucine zipper (bZIP) transcription factor |
| *yrm1* | 1 |  | Zn2-Cys6 zinc-finger transcription factor that activates genes involved in multidrug resistance; paralog of Yrr1p, acting on an overlapping set of target genes |
| *yrr1* | 1 |  | Zn2-Cys6 zinc-finger transcription factor that activates genes involved in multidrug resistance; paralog of Yrm1p, acting on an overlapping set of target genes |
| *zap1* |  |  | Zinc-regulated transcription factor; binds to zinc-responsive promoters to induce transcription of certain genes in presence of zinc, represses other genes in low zinc; regulates its own transcription; contains seven zinc-finger domains |
| **(TOTAL)** | **45** | **33** |  |

**I** – description contains at least one of the following: stress, response, tolerance, resistance;

**II** – description contains at least one of the following: metabol* (including metabolism, metabolic, metabolite), *synthe* (including synthesis, synthetic, biosynthesis, biosynthetic), catabol* (including catabolism, catabolic, catabolite), transport

**Table S2. Growth rates under non-stress and stress conditions from initial measurement (n=1)**

| Strain | Batch | µ_non-stress_ | µ_stress_ | ratio |
| --- | --- | --- | --- | --- |
| *ace2* | 1 | 0.384 | 0.182 | 0.474 |
| *arg80* | 1 | 0.442 | 0.204 | 0.462 |
| *aro80* | 1 | 0.452 | 0.231 | 0.510 |
| *bas1* | 1 | 0.451 | 0.216 | 0.480 |
| *dal80* | 1 | 0.459 | 0.186 | 0.405 |
| *fzf1* | 1 | 0.455 | 0.210 | 0.462 |
| *gcn4* | 1 | 0.458 | 0.168 | 0.367 |
| *gln3* | 1 | 0.460 | 0.216 | 0.469 |
| *gzf3* | 1 | 0.441 | 0.183 | 0.415 |
| *ino4* | 1 | 0.286 | 0.122 | 0.427 |
| *leu3* | 1 | 0.444 | 0.187 | 0.422 |
| *met28* | 1 | 0.438 | 0.180 | 0.410 |
| *mot3* | 1 | 0.466 | 0.204 | 0.438 |
| *oaf1* | 1 | 0.460 | 0.216 | 0.469 |
| *pho4* | 1 | 0.444 | 0.200 | 0.450 |
| *rpn4* | 1 | 0.420 | 0.104 | 0.246 |
| *stp1* | 1 | 0.449 | 0.169 | 0.378 |
| *stp2* | 1 | 0.433 | 0.135 | 0.311 |
| *uga3* | 1 | 0.465 | 0.214 | 0.461 |
| *yap6* | 1 | 0.461 | 0.221 | 0.479 |
| **wt-1** | 1 | 0.457 | 0.160 | 0.351 |
| *arg82* | 2 | 0.401 | 0.100 | 0.249 |
| *bas2/grf10/pho2* | 2 | 0.422 | 0.220 | 0.520 |
| *cad1/yap2* | 2 | 0.443 | 0.248 | 0.560 |
| *dal81* | 2 | 0.439 | 0.244 | 0.557 |
| *hap2* | 2 | 0.433 | 0.214 | 0.495 |
| *hap3* | 2 | 0.413 | 0.171 | 0.415 |
| *hap5* | 2 | 0.444 | 0.227 | 0.512 |
| *ime1* | 2 | 0.423 | 0.231 | 0.545 |
| *ino2* | 2 | 0.244 | 0.155 | 0.634 |
| *ixr1* | 2 | 0.418 | 0.244 | 0.584 |
| *lys14* | 2 | 0.439 | 0.238 | 0.543 |
| *met31* | 2 | 0.396 | 0.222 | 0.561 |
| *opi1* | 2 | 0.264 | 0.156 | 0.593 |
| *thi2* | 2 | 0.434 | 0.236 | 0.543 |
| **wt-2** | 2 | 0.455 | 0.190 | 0.417 |
| *adr1* | 3 | 0.431 | 0.220 | 0.511 |
| *arr1* | 3 | 0.423 | 0.195 | 0.461 |
| *ecm22* | 3 | 0.443 | 0.218 | 0.493 |
| *hac1* | 3 | 0.431 | 0.209 | 0.485 |
| *hap4* | 3 | 0.447 | 0.193 | 0.431 |
| *mig2* | 3 | 0.421 | 0.203 | 0.483 |
| *mks1* | 3 | 0.421 | 0.223 | 0.530 |
| *nrg1* | 3 | 0.414 | 0.209 | 0.504 |
| *pdr1* | 3 | 0.417 | 0.186 | 0.446 |
| *pho23* | 3 | 0.435 | 0.223 | 0.514 |
| *ric1* | 3 | 0.327 | 0.211 | 0.644 |
| *sin3* | 3 | 0.460 | 0.212 | 0.460 |
| *sut1* | 3 | 0.437 | 0.208 | 0.476 |
| *ume6* | 3 | 0.440 | 0.233 | 0.529 |
| **wt-3** | 3 | 0.399 | 0.164 | 0.412 |
| *cat8* | 4 | 0.352 | 0.222 | 0.629 |
| *cha4* | 4 | 0.394 | 0.213 | 0.540 |
| *cin5* | 4 | 0.409 | 0.230 | 0.563 |
| *crz1* | 4 | 0.419 | 0.224 | 0.534 |
| *hal9* | 4 | 0.417 | 0.214 | 0.514 |
| *msn2* | 4 | 0.396 | 0.202 | 0.510 |
| *msn4* | 4 | 0.387 | 0.210 | 0.541 |
| *pdr8* | 4 | 0.434 | 0.251 | 0.578 |
| *ppr1* | 4 | 0.419 | 0.265 | 0.633 |
| *rgt1* | 4 | 0.418 | 0.217 | 0.519 |
| *rim101* | 4 | 0.408 | 0.227 | 0.555 |
| *sip4* | 4 | 0.409 | 0.209 | 0.513 |
| *skn7* | 4 | 0.434 | 0.218 | 0.501 |
| *stb5* | 4 | 0.413 | 0.107 | 0.259 |
| *yap1* | 4 | 0.422 | 0.216 | 0.512 |
| *yap3* | 4 | 0.421 | 0.217 | 0.515 |
| **wt-4** | 4 | 0.410 | 0.202 | 0.493 |
| *aft2* | 5 | 0.463 | 0.237 | 0.513 |
| *arr1* | 5 | 0.439 | 0.205 | 0.468 |
| *gcr2* | 5 | 0.318 | 0.124 | 0.389 |
| *msn1* | 5 | 0.430 | 0.213 | 0.495 |
| *pdr3* | 5 | 0.443 | 0.214 | 0.484 |
| *sfl1* | 5 | 0.455 | 0.183 | 0.401 |
| *sko1* | 5 | 0.428 | 0.272 | 0.635 |
| *swi6* | 5 | 0.377 | 0.076 | 0.201 |
| *tye7* | 5 | 0.453 | 0.249 | 0.550 |
| *usv1* | 5 | 0.445 | 0.216 | 0.486 |
| *wtm2* | 5 | 0.450 | 0.239 | 0.532 |
| *xbp1* | 5 | 0.431 | 0.220 | 0.510 |
| *yap5* | 5 | 0.445 | 0.191 | 0.430 |
| *yap7* | 5 | 0.435 | 0.218 | 0.500 |
| *yrm1* | 5 | 0.454 | 0.205 | 0.452 |
| *yrr1* | 5 | 0.458 | 0.193 | 0.420 |
| **wt-5** | 5 | 0.442 | 0.162 | 0.367 |
| *aft1* | 6 | 0.434 | 0.166 | 0.384 |
| *cbf1* | 6 | 0.349 | 0.166 | 0.476 |
| *cst6* | 6 | 0.476 | 0.219 | 0.460 |
| *gat1* | 6 | 0.468 | 0.245 | 0.523 |
| *gis1* | 6 | 0.476 | 0.257 | 0.540 |
| *mig1* | 6 | 0.441 | 0.222 | 0.505 |
| *mig3* | 6 | 0.471 | 0.254 | 0.539 |
| *put3* | 6 | 0.471 | 0.229 | 0.486 |
| *rsf2* | 6 | 0.456 | 0.183 | 0.402 |
| *zap1* | 6 | 0.448 | 0.224 | 0.498 |
| **wt-6** | 6 | 0.444 | 0.193 | 0.435 |
| *ace1/cup2* | 7 | 0.499 | 0.212 | 0.426 |
| *ada2* | 7 | 0.320 | 0.103 | 0.320 |
| *arg81/argr2* | 7 | 0.477 | 0.214 | 0.449 |
| *azf1* | 7 | 0.491 | 0.281 | 0.573 |
| *cup9* | 7 | 0.457 | 0.202 | 0.442 |
| *dal82* | 7 | 0.471 | 0.232 | 0.494 |
| *gal3* | 7 | 0.492 | 0.217 | 0.440 |
| *gal4* | 7 | 0.508 | 0.209 | 0.411 |
| *gal80* | 7 | 0.466 | 0.216 | 0.464 |
| *gat2* | 7 | 0.474 | 0.245 | 0.518 |
| *mac1* | 7 | 0.448 | 0.240 | 0.537 |
| *mga1* | 7 | 0.507 | 0.168 | 0.331 |
| *ngg1/ada3* | 7 | 0.387 | 0.166 | 0.429 |
| *not3* | 7 | 0.449 | 0.191 | 0.426 |
| *rox1* | 7 | 0.499 | 0.216 | 0.434 |
| *spt23* | 7 | 0.441 | 0.229 | 0.520 |
| *tea1* | 7 | 0.448 | 0.197 | 0.441 |
| **wt-7** | 7 | 0.480 | 0.161 | 0.336 |

Samples labeled **wt-1** to **wt-7** are the BY4742 reference strain samples included in each cultivation batch. ‘ratio’ refers to ratio of µ_stress_/µ_non-stress_.

**Table S3. Specific growth rate measurements under 1.5% (v/v) 1-butanol stress condition for 19 selected strains**

| Strain | Replicate 1 | Replicate 2 | Mean | Standard deviation |
| --- | --- | --- | --- | --- |
| mks1 | 0.1836 | 0.1811 | 0.1823 | 0.0018 |
| sko1 | 0.1724 | 0.1549 | 0.1636 | 0.0124 |
| lys14 | 0.1570 | 0.1532 | 0.1551 | 0.0027 |
| thi2 | 0.1537 | 0.1491 | 0.1514 | 0.0032 |
| leu3 | 0.1432 | 0.1539 | 0.1485 | 0.0076 |
| bas1 | 0.1562 | 0.1407 | 0.1484 | 0.0110 |
| tye7 | 0.1340 | 0.1545 | 0.1443 | 0.0145 |
| gat2 | 0.1429 | 0.1421 | 0.1425 | 0.0006 |
| sip4 | 0.1574 | 0.1267 | 0.1421 | 0.0217 |
| yap6 | 0.1461 | 0.1311 | 0.1386 | 0.0107 |
| azf1 | 0.1340 | 0.1422 | 0.1381 | 0.0058 |
| aro80 | 0.1442 | 0.1319 | 0.1381 | 0.0087 |
| put3 | 0.1455 | 0.1291 | 0.1373 | 0.0116 |
| oaf1 | 0.1436 | 0.1209 | 0.1323 | 0.0160 |
| dal80 | 0.1256 | 0.1269 | 0.1263 | 0.0009 |
| stp2 | 0.1212 | 0.1149 | 0.1181 | 0.0045 |
| gcn4 | 0.1266 | 0.1037 | 0.1152 | 0.0162 |
| rsf2 | 0.1127 | 0.1058 | 0.1092 | 0.0049 |
| mot3 | 0.0714 | 0.0907 | 0.0810 | 0.0136 |
| BY4742 | 0.1198 | 0.1215 | 0.1207 | 0.0012 |

**Table S4. Parameters used in data processing software**

| **MetAlign**  Mass Mode  Mass Bin  Baseline and Noise Peak Elimination Params  Retention Begin (Scan nr)  Retention End (Scan nr)  Maximum Amplitude  Peak Slope Factor (x Noise)  Peak Threshold Factor (x Noise)  Peak Threshold (Abs. Value)  Average Peak Width at Half Height (Scans)  Keep Peak Shape (no alignment)  Pre-Synchronize Scans  Scaling Options  Initial Peak Search Criteria  Begin of 1^st^ Region  End of 1^st^ Region  Begin of 2^nd^ Region  End of 2^nd^ Region  Tuning Alignment Options and Criteria  Alignment Type  Maximum Shift per 100 Scans  Mass Peak Selection  Min. Factor (x Noise)  Min. Nr. of Masses | Nominal  0.60  1  24000  7000000  1  4  200  25  no  no  No Scaling  Scan Nr. Max. Shift  0 20  24000 30  0 0  0 0  Pre-align processing (Iterative)  35  1^st^ Iteration Last Iteration  7 7  10 5 |
| --- | --- |
| **MSClust**  Effective Peaks  Peak Width, scans or time  P.W. Margin Softness  Correlation Threshold  C.T. Margin Softness  PD Reduction  PD Reduction Softness  Stop Criterion | 200  10  2  0.8  0.02  0.8  0.01  2 |
| **AIoutput2**  Peak Table Making  Height Threshold  RT Binning  Peak Identification and Annotation  Available Index  Analysis Type  RI Tolerance  Match Threshold (0.7-0.9)  Filtering  Type  Height Filter  RSD(CV) Filter | 50  2  Retention Index  Non targeted  5  0.8  Accurate  1000  20 |

**Table S5. Target compounds detected in GC/MS analysis**

| Compound Name | RT | RI | RI lib. | RI deviation | QuantMS | Mass similarity |
| --- | --- | --- | --- | --- | --- | --- |
| Unknown | 247.75 | No RI Inf. | - | - | 134 | - |
| Unknown | 248 | No RI Inf. | - | - | 130 | - |
| Unknown | 257.55 | No RI Inf. | - | - | 221 | - |
| n-Propylamine | 282.1 | 1028 | 1028.23 | 0.649 | 174 | 0.993 |
| Oxalacetic acid+Pyruvate | 297.35 | 1050 | 1049.08 | 0.803 | 174 | 0.992 |
| Unknown_2 | 302.15 | 1057 | - | - | 130 | - |
| Lactic acid | 306.75 | 1064 | 1062.36 | 1.270 | 117 | 0.995 |
| Unknown_4 | 312.65 | 1072 | - | - | 221 | - |
| Unknown_5 | 333.55 | 1103 | - | - | 188 | - |
| Alanine_2TMS | 336 | 1107 | 1105.68 | 1.023 | 116 | 0.988 |
| Unknown_6_Organic acid like | 336.05 | 1107 | - | - | 147 | - |
| Unknown_8 | 354.5 | 1135 | - | - | 130 | - |
| Unknown_10 | 366.3 | 1153 | - | - | 191 | - |
| Leucine_1TMS | 369.5 | 1158 | 1157.68 | 0.294 | 86 | 0.999 |
| Unknown_13 | 381.95 | 1177 | - | - | 144 | - |
| Unknown_14 | 409.2 | 1220 | - | - | 130 | - |
| Valine_2TMS_major | 411 | 1223 | 1221.7 | 1.445 | 144 | 0.994 |
| Urea | 419.05 | 1236 | 1236.49 | 0.074 | 147 | 0.996 |
| Unknown_16_Amine like | 422.3 | 1242 | - | - | 174 | - |
| Serine_2TMS_minor | 435.05 | 1263 | 1262.64 | 0.153 | 116 | 0.994 |
| Leucine_2TMS | 445.1 | 1279 | 1278.66 | 0.700 | 158 | 0.994 |
| Unknown_23 | 446.35 | 1281 | - | - | 211 | - |
| Glycerol | 446.7 | 1282 | 1280.81 | 1.188 | 147 | 0.966 |
| Threonine_2TMS_minor | 458.15 | 1301 | 1299.31 | 1.635 | 117 | 0.967 |
| Isoleucine_2TMS_major | 458.5 | 1302 | 1300.64 | 0.930 | 158 | 0.990 |
| Proline_2TMS | 461 | 1306 | 1306.38 | 0.351 | 142 | 0.999 |
| Glycine_3TMS | 466.95 | 1317 | 1316.4 | 0.243 | 174 | 0.996 |
| Uracil | 483.4 | 1346 | 1344.43 | 1.556 | 99 | 0.991 |
| Serine_3TMS_major | 496.75 | 1370 | 1370.14 | 0.340 | 204 | 0.998 |
| Threonine_3TMS_major | 512.45 | 1398 | 1396.08 | 1.726 | 117 | 0.988 |
| Malic acid | 564.45 | 1497 | 1496.36 | 0.455 | 147 | 0.986 |
| Aspartic acid_3TMS | 580.95 | 1530 | 1529.21 | 0.981 | 232 | 0.995 |
| 5-Oxoproline | 582.4 | 1533 | 1532.89 | 0.253 | 156 | 0.981 |
| Glutamic acid_3TMS | 628.65 | 1629 | 1628.11 | 0.803 | 246 | 0.993 |
| Phenylalanine_2TMS | 634.8 | 1642 | 1642.75 | 0.577 | 218 | 0.996 |
| Asparagine_3TMS | 653.55 | 1683 | 1682.41 | 0.190 | 116 | 0.994 |
| Phthalic acid | 668.25 | 1715 | 1707.28 | 7.816 | 147 | 0.823 |
| 2-Aminoadipic acid | 673 | 1726 | 1725.27 | 0.641 | 217 | 0.966 |
| Quinolinic acid | 680 | 1742 | 1742.32 | 0.471 | 147 | 0.922 |
| Ribitol | 684.5 | 1752 | 1753.64 | 1.545 | 217 | 0.978 |
| Orotic acid | 686.6 | 1757 | 1757.76 | 0.884 | 254 | 0.964 |
| Ornithine_3TMS | 691.3 | 1768 | 1768.56 | 0.983 | 174 | 0.959 |
| Glutamine_3TMS | 698.9 | 1785 | 1785.52 | 0.638 | 156 | 0.994 |
| Isocitric acid+Citric acid | 721.75 | 1839 | 1837.41 | 1.370 | 147 | 0.968 |
| 4-Aminobenzoic acid | 723.4 | 1843 | 1843.24 | 0.513 | 192 | 0.970 |
| Cadaverine | 729.3 | 1857 | 1856.9 | 0.058 | 174 | 0.988 |
| Glucose_1 | 760.85 | 1934 | 1932.24 | 1.722 | 205 | 0.956 |
| Lysine_4TMS | 763.35 | 1940 | 1939.94 | 0.306 | 156 | 0.968 |
| Galactose_2+Glucose_2 | 768.75 | 1954 | 1950.45 | 3.371 | 147 | 0.931 |
| Tyrosine | 770.45 | 1958 | 1956.8 | 1.295 | 218 | 0.993 |
| Sorbitol | 777.7 | 1976 | 1977.61 | 1.290 | 205 | 0.944 |
| Glucarate_1_same | 781.1 | 1985 | 1980.9 | 3.967 | 217 | 0.874 |
| Plamitic acid(16:0) | 804.85 | 2047 | 2047.65 | 1.041 | 117 | 0.966 |
| Inositol | 837.25 | 2133 | 2132.25 | 1.019 | 217 | 0.961 |
| Tryptophan_1TMS | 867.5 | 2217 | 2217.35 | 0.148 | 218 | 0.997 |
| Tryptamine_1 | 873.3 | 2234 | 2233.05 | 0.780 | 128 | 0.984 |
| Stearic acid(17:0) | 876.9 | 2244 | 2244.1 | 0.051 | 117 | 0.950 |
| Tryptophan_3TMS | 878.85 | 2250 | 2249.15 | 0.592 | 202 | 0.997 |
| Spermidine | 891.65 | 2286 | 2285.18 | 1.259 | 144 | 0.941 |
| Cystine | 904.05 | 2323 | 2324.78 | 1.843 | 218 | 0.912 |
| Fructose 6-phosphate_2 | 915.2 | 2356 | 2355.34 | 0.940 | 315 | 0.895 |
| Fructose 6-phosphate_3 | 920.15 | 2371 | 2370.11 | 0.973 | 387 | 0.928 |
| Unknown_98 | 942.65 | 2440 | - | - | 150 | - |
| Sucrose | 1024.4 | 2706 | 2706.2 | 0.432 | 361 | 0.920 |
| Trehalose | 1056.35 | 2817 | 2816.81 | 0.552 | 191 | 0.949 |
| Unknown_102 | 1108.3 | 3008 | - | - | 105 | - |

Table compiled with information from the PeakTableUpdate and IdentificationTable worksheets in AIoutput2.

**Table S6. Specific growth rates under 1.5% (v/v) 1-butanol stress condition for new strains**

| Strain | Replicate 1 | Replicate 2 | Replicate 3 | Replicate 4 | Average | Standard deviation | *p*-value |
| --- | --- | --- | --- | --- | --- | --- | --- |
| his3 | 0.1329 | 0.1280 | 0.1565 | 0.1378 | 0.1388 | 0.0124 | 0.5000 |
| cha1 | 0.1558 | 0.1503 | 0.1526 |  | 0.1529 | 0.0028 | 0.0526 |
| met2 | 0.1696 | 0.1635 | 0.1646 |  | 0.1659 | 0.0033 | 0.0091 |
| aat1 | 0.1456 | 0.1327 | 0.1463 | 0.1527 | 0.1443 | 0.0084 | 0.2482 |
| aat2 | 0.1523 | 0.1640 | 0.1451 | 0.1421 | 0.1509 | 0.0098 | 0.0906 |
| cit1 | 0.1378 | 0.1553 | 0.1508 | 0.1433 | 0.1468 | 0.0078 | 0.1625 |
| cit2 | 0.1720 | 0.1873 | 0.1787 | 0.1816 | 0.1799 | 0.0064 | 0.0015 |
| cit3 | 0.1332 | 0.1420 | 0.1398 | 0.1519 | 0.1417 | 0.0077 | 0.3545 |
